# Application of electron beam water radiolysis for sewage sludge treatment—a review

**DOI:** 10.1007/s11356-020-10643-0

**Published:** 2020-09-06

**Authors:** Malgorzata Siwek, Thomas Edgecock

**Affiliations:** University of Huddersfield, HD13DH, Queensgate, Huddersfield, West Yorkshire UK

**Keywords:** Electron beam irradiation, Sewage sludge treatment, Water radiolysis, *G* value, Free radical scavengers, Electron penetration depth, Wastewater hygienisation

## Abstract

A review of the applicability of electron beam water radiolysis for sewage sludge treatment is presented. Electron beam treatment has been proven to be a successful approach to the disinfection of both wastewater and sewage sludge. Nevertheless, before 2000, there were concerns about the perceived high capital costs of the accelerator and with public acceptance of the usage of radiation for water treatment purposes. Nowadays, with increased knowledge and technological development, it may be not only possible but also desirable to use electron beam technology for risk-free sewage sludge treatment, disposal and bio-friendly fertiliser production. Despite the developing interest in this method, there has been no attempt to perform a review of the pertinent literature relating to this technology. It appears that understanding of the mechanism and primary parameters of disinfection is key to optimising the process. This paper aims to reliably characterise the sewage sludge electron beam treatment process to elucidate its major issues and make recommendations for further development and research.

**Figure:**
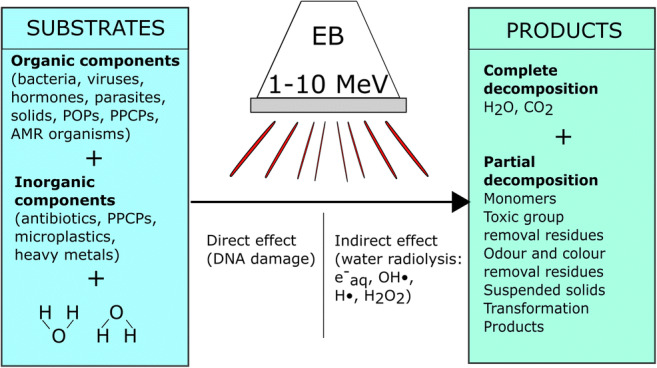
Graphical abstract

Graphical abstract

## Historical background

The interest in using accelerated electrons for sewage and sewage sludge treatment has been rising for several decades, and the earliest large-scale studies were started in North America in 1974 (Trump et al. 1984). This led to experiments with a first pilot plant system at the Deer Island Wastewater Treatment Plant in Boston. There was also a research plan undertaken in the Miami-Dade Virginia Key Wastewater Treatment Plant in 1976. The Electron Beam Research Facility (EBRF) was built, and it operated for 1 year (Waite et al. 1985). Extensive industrial-scale research on the application of electron beams for the spent water treatment was conducted in EBRF in 2000 (Kurucz et al. 1995; Nickelsen et al. 1994; Tobien et al. 2000; Waite et al. 1985). There was also an investigation of the acceleration technology efficiency in the sewage treatment plant in Kolo, Poland, in cooperation with the Institute of Nuclear Chemistry and Technology in Warsaw (Chmielewski et al. 1995).

The largest commercial venture was the removal of dye from wastewater at the Daegu Dyeing Industrial Complex (DDIC) in Korea. After the construction of a pilot plant, the purification of wastewater from this textile dyeing was applied commercially from 2005 (Han et al. 2012).

Apart from industrial pilot plants, there have also been numerous research studies of electron beam (EB) treatment on the laboratory scale. These fundamental studies have revealed that EB has a profound influence on bacteria, virus and parasite removal (Borrely et al. 1998; Capodaglio 2017; Chmielewski et al. 1995; Engohang-Ndong et al. 2015; Farooq et al. 1992; Getof 1995; Hossain et al. 2018; Maruthi et al. 2013, Maruthi et al. 2011a; Moraes et al. 2004; Praveen et al. 2013; Pribil et al. 2007; Rawat and Sarma 2013) and the solubility of carbohydrates, proteins and lipids (Changqing and Min 2012; Engohang-Ndong et al. 2015; Lim et al. 2016; Park et al. 2009; Shin and Kang 2003), as well as colour and odour removal (Bae et al. 1999; Engohang-Ndong et al. 2015; Kim et al. 2007; Paul et al. 2011; Tobien et al. 2000). Another crucial issue is the electron beam irradiation effect on biochemical oxygen demand (BOD) and chemical oxygen demand (COD), which are used worldwide to measure the number of organic compounds in water (Royal Commission on Sewage Disposal 1915; U.S. Environmental Protection Agency 2011). Many papers claim that BOD and soluble COD increase after penetration by accelerated electrons (Changqing and Min 2012; Han et al. 2012; Kim et al. 2007; Lim et al. 2016; Park et al. 2009; Paul et al. 2011; Shin and Kang 2003), which is thought to be caused by the conversion of non-biodegradable compounds into biodegradable form. Nonetheless, several others say that BOD and COD decrease after EB irradiation (Bae et al. 1999; Farooq et al. 1992; He et al. 2016; Maruthi et al. 2011b; Rawat and Sarma 2013; Zheng et al. 2001), which might be a result of the breakdown of biodegradable components into simple, harmless products such as water, carbon oxides and salts (Cross and Jayaram 1998; Son 2017). Therefore, a closer look at the literature reveals complications in understanding the mechanisms of pollutant removal from the spent water after electron beam application and the need for a review.

## The mechanism of the electron beam irradiation

### Sewage sludge direct and indirect radiolysis

The microbicidal action of ionising radiation (IR) is achieved by its direct (physical) and indirect (chemical) action. When the high energy electrons interact with sewage sludge matter, the ionisation and excitation of molecules can occur (Borrely et al. 1998):

- R. 1:*XY* + *e* ^−^ ➔ *XY* ^∗^ + *XY* ^+^ + *e*^−^
- R. 2:*XY* ^+^ + *e* ^−^ ➔ *XY*^∗^
- R. 3:*XY*^ ∗^ ➔ *X* • + *Y*•
- R. 4:*XY*^ +^ ➔ *X* ^+^ + *Y*•

Where R. 1—electronic excitation and ionisation, R. 2—recombination, R. 3 and R. 4—fragmentation, ^*^—excited molecule, ^+/−^—cation/anion and •—radical.

Once the irradiation is applied, the electrons can interact with the genetic material or some other cellular elements that are essential to the persistence of the organism. This is called a direct effect, and it may ultimately affect the ability of the cell to reproduce and survive. The direct effect of the radiation is thought to play a small part in treating pathogens (Lemée et al. 2017) and may only be significant, > 10% of removal, with organic compounds when the concentration of the contaminant is ≥ 0.1 M (William et al. 2001).

As a result of water radiolysis, several reactive species are formed which can further interact with each other and with the components of the sewage sludge (Cross and Jayaram 1998). This is called an indirect effect. During the water treatment, free radicals and hydrogen peroxide might be generated directly in the biological cell, resulting in its damage or permanent impairment. The biological material destruction efficiency is correlated with the amount of energy absorbed. Actively reducing reactive components (e^−^_aq_, hydrated electron, and H•, hydrogen radical) and strongly oxidising radical (OH•, hydroxyl radical) are formed all but simultaneously, within 10^−7^ s of exposure, and in the same order of magnitude concentration (Table 2). This is beneficial to degrade mixed contaminants that could be removed via either oxidation or reduction and that aspect differentiates the EB process from other advanced oxidation technologies (Wang and Chu 2016). The reactions that take place are given in Table 1, along with their rate constants.

**Table 1:** *Reactions occurring in pure water after the electron beam irradiation and corresponding rate constants according to different authors*

Reaction	Rate constant [mol^−1^ dm^3^ s^−1^]	Reference
H• + O_2_•^**−**^ ➔ HO_2_•^−^	2.0 × 10^10^	William et al. (2001)
H• + H_2_O_2_ ➔ H_2_O + OH•	9.0 × 10^7^	Buxton et al. (1988), William et al. (2001)
H• + O_2_ ➔ HO_2_•	2.1 × 10^10^	William et al. (2001)
H• + H• ➔ H_2_	7.8 × 10^9^	Le Caër (2011)
	5.0 × 10^9^	William et al. (2001)
	1.3 × 10^10^	Sun and Chmielewski (2017)
H• + H_2_O ➔ H_2_ + OH•	1 × 10^1^	Buxton et al. (1988), William et al. (2001)
H• + HO_2_• ➔ H_2_O_2_	1.0 × 10^10^	William et al. (2001)
H• + OH^−^ ➔ e^−^_aq_ + H_2_O	2.2 × 10^7^	William et al. (2001)
e^−^_aq_ + H_2_O_2_ ➔ OH• + OH^−^	1.2 × 10^10^	Kurucz et al. (1991), William et al. (2001)
	1.1 × 10^10^	Buxton et al. (1988)
e^−^_aq_ + OH• ➔ OH^−^	3.0 × 10^10^	Buxton et al. (1988), Khan et al. (2015), Le Caër (2011), William et al. (2001)
e^−^_aq_ + H^+^ ➔ H•	2.3 × 10^10^	Buxton et al. (1988), Tobien et al. (2000), Wang and Chu (2016)
e^−^_aq_ + H_3_O^+^ ➔ H• + H_2_O	2.3 × 10^10^	Le Caër (2011)
e^−^_aq_ + e _aq_ + 2H_2_O ➔ H_2_ + 2OH^−^	5.5 × 10^9^	Buxton et al. (1988), Le Caër (2011)
e^−^_aq_ + H• + H_2_O ➔ H_2_ + OH^−^	2.5 × 10^10^	Le Caër (2011)
e^−^_aq_ + H_2_O ➔ H• + OH^−^	1.9 × 10^1^	Buxton et al. (1988)
	8.9 × 10^2^	William et al. (2001)
e^−^_aq_ + H• ➔ H_2_ + OH^−^	2.5 × 10^10^	Buxton et al. (1988)
e^−^_aq_ + O•^−^ ➔ 2OH^−^	2.2 × 10^10^	Buxton et al. (1988), William et al. (2001)
e^−^_aq_ + HO_2_^−^ ➔ 2OH^−^ + OH•	3.5 × 10^9^	William et al. (2001)
e^−^_aq_ + O_2_ ➔ O_2_•^**−**^	1.9 × 10^10^	Buxton et al. (1988), Getof (1995), William et al. (2001)
e^−^_aq_ + O_2_^**−**^• ➔ O_2_^2−^	1.3 × 10^10^	Buxton et al. (1988)
OH• + H_2_ ➔ H• + H_2_O	4.2 × 10^7^	Buxton et al. (1988)
OH• + OH• ➔ H_2_O_2_	5.5 × 10^9^	Khan et al. (2015), Le Caër (2011)
OH• + HO_2_• ➔ H_2_O + O_2_	6.0 × 10^9^	Buxton et al. (1988)
OH• + H• ➔ H_2_O	7.0 × 10^9^	Buxton et al. (1988), Khan et al. (2015), Wang and Chu (2016)
	2.0 × 10^10^	Le Caër (2011)
OH• + OH^−^ ➔ O•^**−**^ + H_2_O	1.2 × 10^10^	Wang and Chu (2016)
	1.3 × 10^10^	Buxton et al. (1988), William et al. (2001)
OH• + HO_2_^−^ ➔ H_2_O + O_2_•^**−**^	7.5 × 10^9^	William et al. (2001)
OH• + O_2_•^**−**^ ➔ OH^−^ + O_2_	8 × 10^9^	Buxton et al. (1988)
	1.1 × 10^10^	William et al. (2001)
O• + H_2_O_2_ ➔ O_2_•^**−**^ **+** H_2_O	2.7 × 10^7^	William et al. (2001)
O•^**−**^ **+** H_2_O ➔ OH^−^ + OH•	1.8 × 10^6^	Buxton et al. (1988)
O•^**−**^ **+** H_2_ ➔ H• + OH^−^	8.0 × 10^7^	Buxton et al. (1988)
O•^**−**^ **+** HO_2_^**−**^ ➔ O_2_•^**−**^ **+** OH^**−**^	4.0 × 10^8^	Buxton et al. (1988)
O•^**−**^ **+** O_2_•^**−**^ ➔ 2OH^**−**^ + O_2_	6.0 × 10^8^	William et al. (2001)
H_3_O^+^ + OH^−^ ➔ 2H_2_O	1.4 × 10^11^	Le Caër (2011), Sun and Chmielewski (2017)
HO_2_• + O_2_•^**−**^ **➔** H_2_O_2_ + O_2_ + OH^−^	9.7 × 10^7^	William et al. (2001)
HO_2_• + HO_2_• **➔** H_2_O_2_ + O_2_	8.3 × 10^5^	William et al. (2001)

The primary species H•, OH• and e^−^_aq_, as well as H_2_O_2_, can penetrate the organic molecules, and they are the most reactive.

### *G* value for water irradiation-induced reactive species

In order to determine the number of radicals produced, the *G* parameter can be used. *G* radiation chemical yield (1) is the number of molecules, atoms or free radicals created (or destroyed) per 100 eV of energy deposited in water (Buxton et al. 1988).

1 $$ G=\frac{No.\kern0.5em of\ formed\ molecules}{100\  eV} $$

The main reactive compounds formed after the injection of electrons into the water and their *G* values (neutral conditions) are as follows (Cross and Jayaram 1998; Engohang-Ndong et al. 2015; Nickelsen et al. 1994; Tobien et al. 2000; Wang and Chu 2016):

- R. 5:H_2_O ➔ [2.7] OH• + [2.6] e^−^_aq_ + [0.6] H• + [2.6] H_3_O^+^ + [0.45] H_2_ + [0.7] H_2_O_2_

Using the *G* value in the SI unit of μmol of product formed (or destroyed) after the absorption of 1 J of energy, the approximate concentration of the reactive species can be evaluated (Cooper et al. 2004):

2 $$ {C}_{RC}=D\times {G}_{\mathrm{valueRC}} $$

Where C_RC_—reactive compound concentration [μmol /kg], D—applied dose [J /kg] and G_valueRC_—G value of the reactive compound [μmol /J]. This can be calculated by multiplying the values in R. 5 by 0.1036.

The approximate content of radicals, hydrogen peroxide and the hydrated electron in pure water at various doses using high energy electron acceleration is presented in Table 2 (Cooper et al. 2004).

**Table 2:** The estimated concentration of reactive components at various doses using the electron beam (based on Cooper et al. 2004)

Dose [kGy]	Concentration [mM]			
	e^−^_aq_	H•	OH•	H_2_O_2_
1	0.27	0.06	0.28	0.07
5	1.4	0.3	1.4	0.4
10	2.7	0.6	2.8	0.7
15	4.1	0.9	4.2	1

As the dose increases, such a simple estimation may no longer be strictly accurate, and the concentration might be over-estimated (the contribution of direct effect increases). However, in the highly contaminated environment of sewage sludge, where the reactive species action is being rapidly inhibited, this assumption provides an order-of-magnitude estimation of the compounds available for reaction with the organic or inorganic contamination (William et al. 2001). An approximate removal percentage can be calculated for each species (3) (Nickelsen et al. 1994). As an example, the estimated e^−^_aq_, H• and OH• contributions in toluene and benzene removal are presented in Table 3.

3 $$ {C}_x=\frac{k_x\times {G}_x\times 100\%}{k_x\times {G}_x+{k}_y\times {G}_y+{k}_z\times {G}_z} $$

**Table 3:** The approximate responsibility of each reactive species for toluene and benzene removal (based on Nickelsen et al. 1994)

Component	Contribution in disinfection effect [%]		
	e^−^_aq_	H•	OH•
Toluene	0.1	2.5	97.4
Benzene	0.4	16.1	83.5

Where *k*_*x*,*y*,*z*_ are rate constants [mol^−1^ dm^3^ s^−1^] of reactions between the contaminant and the *x*,*y*,*z* reactive species; *G*_*x*,*y*,*z*_ are the *G* values of *x*,*y*,*z* radical formation; and *C*_*x*_ is the *x* radical contribution in contaminant removal.

Using the *G* value allows the identification of the most substantial radicals for specific contaminants and should be one of the first steps when designing wastewater or sewage sludge electron beam installation, where specific contaminants are present.

### Free radical scavengers

In order to extend the laboratory data to natural conditions, the composition of sewage sludge should be considered. Nonetheless, while thousands of experiments have been carried out on the radiolytic destruction of various groups of environmental contaminants, these were tested mostly on single-component, synthetic solutions. In real conditions (sewage sludge, wastewater/natural water with a complex matrix), many constituents naturally present in solution were observed to scavenge the production of the reactive chemical species during electron beam irradiation (e.g. O_2_, HCO_3_^-;^, CO_3_^2−^, Cl^−^, NO_2_^-^, NO_3_^-^) decreasing or increasing the overall process efficiency (Capodaglio 2017; Wang and Chu 2016; William et al. 2001).

Scavengers are defined as chemical compounds (or ions and radicals), which react with reactive species produced by radiolysis. Such additional, both organic and inorganic, chemicals can compete with the target pollutant. Some well-known natural water scavengers are oxygen, bicarbonate/carbonate ions, and nitrate ions as well as dissolved organic carbon (DOC) (Wang and Chu 2016; William et al. 2001) or heavy metal (HM) ions (Duarte et al. 2004; William et al. 2001). Oxygen is reduced by rapid reaction with both e^−^_aq_ and H•, while nitrate ions act as an e^−^_aq_ scavenger, and during radiolysis, they are reduced to nitrite ions. The NO_2_^−^ can further react with the OH• and this promotes the addition of NO_2_ to aromatic solutes if present (Wang and Chu 2016; William et al. 2001). The carbonate ion is an ascertained hydroxyl radical scavenger, and both carbonate and bicarbonate ions are used for the alkalinity estimation of natural systems. However, the scavenging effect of alkalinity on OH• radical is strictly dependent upon the solution pH (William et al. 2001). This phenomenon is described in detail in the “pH influence” section. The metal concentration is also a parameter of high importance. It has been reported that some of the main products of water irradiation are scavenged by the metal ions (Duarte et al. 2004). As described in “Heavy metal influence and removal” section, this makes it possible to use the electron beam for HM removal.

Rate constants of reactions between chemical species typically found in natural water (nitrate, nitrite, carbonate, bicarbonate ions and oxygen) and reactive components created during the water radiolysis are listed in Table 4.

**Table 4:** Examples of chemical reactions and rate constants for various scavengers

Scavenger	Reaction	Rate constant [mol^−1^ dm^3^ s^−1^]	Reference
Carbonate ion	CO_3_^2−^ + OH• ➔ CO_3_•^−^ + OH	3.9 × 10^8^	(Buxton et al. (1988), Nickelsen et al. (1994), Wang and Chu (2016)
	CO_3_^2−^ + e^−^_aq_ ➔ PDTS	3.5 × 10^5^	William et al. (2001)
Bicarbonate ion	HCO_3_^−^ + OH• ➔ CO_3_•^−^ + H_2_O	8.5 × 10^6^	Nasseri et al. (2017), Nickelsen et al. (1994), Wang and Chu (2016)
	HCO_3_^−^ + H• ➔ PDTS	4.4 × 10^4^	William et al. (2001)
	HCO_3_^−^ + e^−^_aq_ ➔ PDTS	1.0 × 10^6^	William et al. (2001)
Nitrite ion	NO_2_^−^ + OH• ➔ NO_2_• + OH^−^	8.0 × 10^9^	Wang and Chu (2016)
	NO_2_^−^ + e^−^_aq_ ➔ NO_2_•^−^	3.5 × 10^9^	Wang and Chu (2016)
Nitrate ion	NO_3_^−^ + e^−^_aq_ ➔ NO_3_•^2−^	9.7 × 10^9^	Buxton et al. (1988), Wang and Chu (2016), William et al. (2001)
	NO_3_^−^ + H• ➔ PDTS	1.4 × 10^6^	William et al. (2001)
DOC	DOC + e^−^_aq_ ➔ PDTS	1.0 × 10^7^	William et al. (2001)
	DOC + OH• ➔ PDTS	1.0 × 10^7^	William et al. (2001)
	DOC + H• ➔ PDTS	1.0 × 10^8^	William et al. (2001)
Methanol	CH_3_OH + OH• ➔ H_2_O + CH_2_OH• (93%) + CH_3_O• (7%)	9.7 × 10^8^	Buxton et al. (1988), Nickelsen et al. (1994), Wang and Chu (2016)
	CH_3_OH + e^−^_aq_ ➔ H• + CH_3_O^−^	< 1.0 × 10^4^	Wang and Chu (2016)
	CH_3_OH + H• ➔ H_2_ + CH_2_OH•	2.6 × 10^6^	Nickelsen et al. (1994)
*t*-BuOH	C_4_H_9_OH + OH• ➔ H_2_O + C_4_H_8_OH•	6.0 × 10^8^	Buxton et al. (1988), Tobien et al. (2000), Wang and Chu (2016), Wojnarovits et al. (2005)
		3.8–7.6 × 10^8^	Nasseri et al. (2017)
	C_4_H_9_OH + e^−^_aq_ ➔ H• + C_4_H_9_O^−^	4.0 × 10^5^	Wang and Chu (2016)
	C_4_H_9_OH + H• ➔ H_2_ + C_4_H_8_OH•	8.0 × 10^4^	Zona et al. (2008)
		10^5^	Buxton et al. (1988)
Thiourea	H_2_NCSNH_2_ + OH• ➔ PDTS	3.9 × 10^9^	Wang and Chu (2016)
	H_2_NCSNH_2_ + e^−^_aq_ ➔ PDTS	2.9 × 10^9^	Wang and Chu (2016)
Isopropanol	C_3_H_7_OH + OH• ➔ H_2_O + C_3_H_6_OH•	1.9 × 10^9^	Wang and Chu (2016)
	C_3_H_7_OH + H• ➔ H_2_ + C_3_H_6_OH•	7.4 × 10^7^	Wang and Chu (2016)
Nitrous oxide	N_2_O + H• ➔ OH• + N_2_	2.1 × 10^9^	Wang and Chu (2016)
	N_2_O + e^−^_aq_ + H_2_O ➔ OH• + N_2_ + OH^−^	9.1 × 10^9^	Buxton et al. (1988), Getof (1995), Tobien et al. (2000), Wang and Chu (2016), Wojnarovits et al. (2005)
Chloride ion	Cl^−^ + OH• ➔ ClOH•^−^	4.3 × 10^9^	Wang and Chu (2016)
		6.1(+/− 0.8) × 10^9^	Nasseri et al. (2017)
Sulfate ion	SO_4_^2−^ + e^−^_aq_ ➔ SO_4_^3−^	1.0 × 10^6^	Wang and Chu (2016)
Bromide ion	Br^-^ + H• ➔ PDTS	2.8 × 10^7^	William et al. (2001)
	Br^-^ + OH• ➔ PDTS	1.1 × 10^10^	William et al. (2001)
Chloramine	NH_2_Cl + e^−^_aq_ ➔ PDTS	1.0 × 10^8^	William et al. (2001)
	NH_2_Cl + OH• ➔ PDTS	1.0 × 10^8^	William et al. (2001)
Ferrocyanide ion	Fe (CN)_6_^4−^ + OH• ➔ Fe (CN)_6_^3−^ + OH^−^	1.1 × 10^10^	Buxton et al. (1988)
Chloroacetic acid	ClC_2_H_3_O_2_ + H• ➔ C_2_H_2_ClO_2_ + H_2_	1.8 × 10^5^	Stadlbauer et al. (1997)
	ClC_2_H_3_O_2_ + e^−^_aq_ ➔ C_2_H_3_O_2_• + Cl^−^	1.9 × 10^9^	Buxton et al. (1988)
Oxygen	O_2_ + e^−^_aq_ ➔ O_2_•^**−**^	1.9 × 10^10^	Buxton et al. (1988), William et al. (2001)
	O_2_ + H• ➔ HO_2_•	2.1 × 10^10^	William et al. (2001)

*PDTS* products

Scavengers are mostly known as harmful components, which can react with radicals and make them no longer available for the contamination removal. While the negative aspect of scavenger occurrence is indisputable, the benefit from its presence might not be as evident. After the identification of a potential reactive species, which is responsible for removing the pollution of interest, it is necessary to study its main reactions with other radicals. It may be favourable to remove expendable reactive species using scavengers, for overall efficiency improvement. For instance, if the destruction of CCl_4_, which reacts with hydrated electron is an objective, one way to raise the removal efficiency (the effective concentration of e^−^_aq_) is to dispose of OH•, because the hydroxyl radical reacts with e^−^_aq_ (Table 1), but does not react with carbon tetrachloride (International Atomic Energy Agency 2005; William et al. 2001). Such a procedure would leave a hydrated electron to react with the contaminant, and the removal efficiency is increased. The same course of action may be repeated for any radical–pollutant pair, and after a deep investigation of sewage sludge components, the presence of scavengers may be used to improve efficacy.

Nonetheless, the sewage sludge composition is often the main problem when considering the strengthening of the positive scavengers’ influence or their detrimental effect reduction. In the past two decades, the numerous, emerging contaminants have been detected in wastewaters and their sludge, such as persistent organic pollutants (POPs) (pesticides, industrial chemicals, chemical by-products like hexachlorobenzene, polychlorinated dibenzofurans) (Changqing and Min 2012), anti-inflammatory drugs and antibiotics (Wang and Chu 2016) as well as antibiotic-resistant genes (Liao Yinguang Chen 2018) and microplastics (Carr et al. 2016; Eckert et al. 2018; Gies et al. 2018; Lasee et al. 2017; Murphy et al. 2016; Prata 2018; Talvitie et al. 2017; Ziajahromi et al. 2017). The electron beam is proven to be efficient for the reduction of 4-chlorophenol (International Atomic Energy Agency 2005), chloroform, dichloroethane, methyl isobutyl ketone, xylene and phenol (Duarte et al. 2004, 2002), pesticides, polycyclic aromatic hydrocarbons (PAHs) and polychlorinated biphenyls (PCBs) (Changqing and Min 2012), various azo dyes (Han et al. 2012, 2002; He et al. 2016; Kim et al. 2006; Paul et al. 2011; Takács et al. 2007; Wojnárovits and Takács 2008), benzene (Duarte et al. 2004; Gholami et al. 2014; Nickelsen et al. 1994), toluene (Duarte et al. 2004, 2002; Nickelsen et al. 1994) and trichloroethylene (Cross and Jayaram 1998) as well as personal and pharmaceutical care products (PPCPs) and many others. However, when the contaminants’ content is very high (i.e. industrial discharges), the initial radical attack results in the by-products’ formation, known as transformation products. These by-products may consequently react with the free radicals and play a role of the scavenger by competition with the pollutant of interest. Therefore, as the treatment proceeds (or dose increases), the removal efficiency may decrease. For low concentrated solutions, the process more likely destroys the reaction by-products along with the initial solute; hence, the removal efficiency increases with time and with higher doses (William et al. 2001). Nevertheless, since the likelihood of by-product occurrence is relatively high due to the complexity of the sewage sludge matrix, the content of the mentioned, dangerous contaminants should always be considered. Examples of chemical reactions between reactive species and various scavengers as well as their rate constants (in pure water) are listed in Table 4. However, the more complicated the wastewater and sewage sludge matrix is, the more elaborate the assessment of scavenging influence, because it may be affected by the presence of different chemicals.

### pH influence

**Fig. 1:**
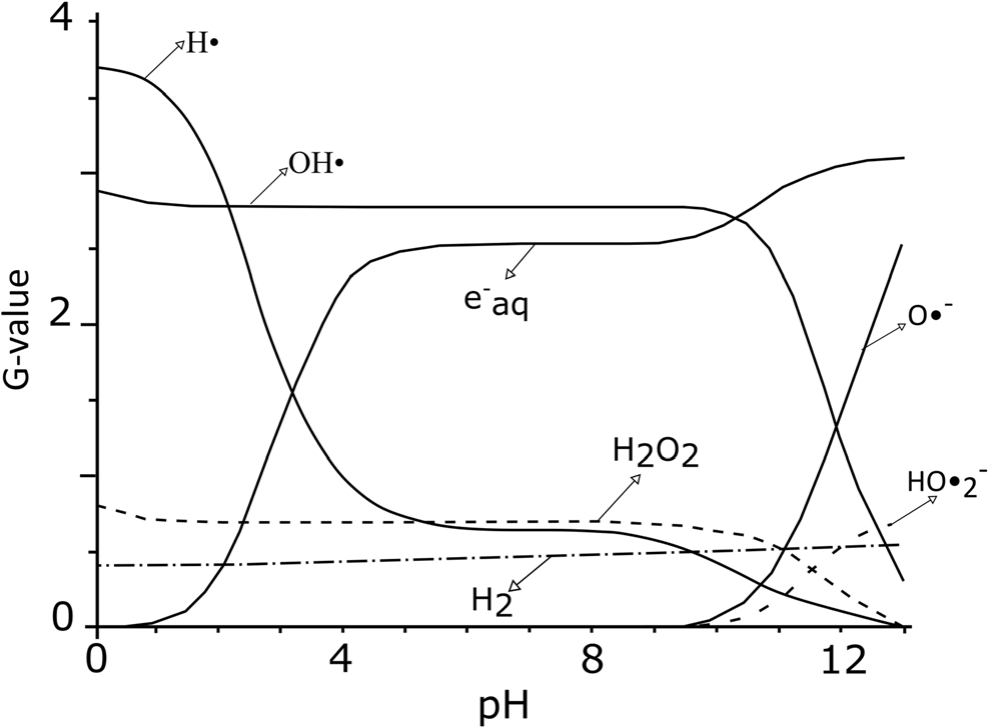
The pH value impact on the main water radiolysis products. *G* values of the primary reactive species formed after the electron beam water irradiation *is shown as a function of pH (based on* Getof 1995*;* Wang and Chu 2016*;* Zona et al. 2008*)*

The electron beam-induced decomposition of organic and inorganic pollutants is a result of their reaction with the water treatment products, such as e^−^_aq_, H• and OH•. Since radicals and hydrated electron can also react with the products of water hydrolysis (H_3_O^+^, OH^−^), the pH value can significantly change their initial yields (*G* values), as shown below (Fig. 1).

At acid conditions, e^−^_eq_ might react with hydrogen ion to produce a hydrogen radical (Table 1), which most likely leads to the radical recombination reaction (Wang and Chu 2016). Therefore, the amount of OH• radicals is reduced. At alkaline pH, the highly oxidising OH• transforms readily to less reactive O•^−^ radical (Buxton et al. 1988; Wang and Chu 2016; William et al. 2001). It is claimed (William et al. 2001) that the scavenging effect on hydroxyl radicals in untreated water (pH 9) is 2.5 times higher than in secondary wastewater (pH 7). It is well documented that pollutant destruction efficiency is profoundly lower in alkaline conditions. Likewise, in many cases, the degradation of contaminant decreases in strong acid conditions (generally pH < 2.0), but it is favoured at weak acid to neutral conditions (Bae et al. 1999; Getof 1995; Nickelsen et al. 1994; Park et al. 2009; Paul et al. 2011; Shin and Kang 2003; Tobien et al. 2000; Wang and Chu 2016; William et al. 2001; Zona et al. 2008).

It can be seen (Fig. 1) that the range of interest for highly productive treatment (e^−^_aq_, OH•) is relatively wide. The number of priority reactive species is significant at pH between 4 and 10. Regarding the complex composition of sewage sludge, the presence of both oxidising (OH•) and reducing (e^−^_aq_) elements is strongly desirable. A closer look at the literature reveals that the general assessment of the most beneficial environment (pH) for electron beam sewage sludge irradiation has not been done so far. Nevertheless, the chemistry of such waste should always be considered first. The constitution of sludge or wastewater of different origins may require the use of different radicals. Thus, controlling of solution pH is a parameter, which may relatively easily increase the electron beam treatment efficiency.

### Heavy metal influence and removal

Since the heavy metals can react with the water radiolysis products, they are known as scavengers. Simultaneously, it is possible to use the EB installation to remove HMs from liquid waste. The method is based on the radiation chemical reduction of the metal ions to their respective metals or to lower oxidation state ions which can then be removed by filtration (Pikaev et al. 1997; Schmelling et al. 1998). The reduction upon electron beam treatment can be a result of reactions of the ions with hydrated electrons e^−^_aq_ and H• atoms, formed from water radiolysis. There are two requirements for heavy metal removal to take place: the absence of oxygen in the water (in the case of Cd^II^ and Pb^II^) and scavenging of OH• radicals (e.g. by formate presence), which can oxidise the reduced metal ions (Pikaev et al. 1997). Since the hydroxyl radicals are the main components responsible for chemical and biological contaminant destruction, it is not possible to remove the HMs simultaneously with the other contaminants.

### Influence of the presence of pathogens

There have been multiple pathogens detected in sludge samples, and their presence is mostly dependent on sludge origin (whether industrial, pharmaceutical or municipal) and geographical location (Maruthi et al. 2013; Tell et al. 2019). The list of the most common pathogens that have been found in the raw sludge can be seen in Table 5.

**Table 5:** List of pathogens that can be found in raw sludge (adapted from Skowron et al. 2018)

Pathogen type	Detected group	Pathogen type	Detected group
Bacteria	Coliforms Faecal coliforms Faecal streptococci Enterococci *Clostridium perfringens* *Staphylococcus* (coagulase positive) *Pseudomonas aeruginosa* Acid-fast bacteria Coliphages *Bacteroides*	Viruses	Adenovirus, Alfavirus (African swine fever) Enterovirus Herpesvirus Parvovirus Picornavirus (foot and mouth disease) Reoviridae Rinovirus
Fungi	*Candida* spp. *Cryptococcus* spp. *Geotrichum* spp. *Rhodotorula* spp. *Trichosporon* spp. *Torulopsis* spp. *Mucor* spp. *Penicillium* spp. *Aspergillus* spp. *Botryotrichum* spp.	Parasites	*Trichostrongylus colubriformis* *Cooperia punctata* *Fasciola hepatica* *Toxocara vituloru* *Ascaris* spp. *Taenia* spp. *Strongylus* spp. *Dictyocaulus* spp. *Dicrocoelium* spp. *Moniezia* spp. *Oesophagostomum* spp.
Protozoa	*Giardia* spp. *Cryptosporidium* spp. *Eimeria* spp. *Balantidium* spp.		

Amongst all of the pathogen groups, there are several species of particular importance due to the severity of the disease they cause or their infectiousness and prevalence. The summary of the most significant representatives is shown in Table 6.

**Table 6:** Pathogens of the most prevalence, infectiousness or importance found in sludge

Pathogen	Disease caused	Additional information	Reference
*E. coli* strains (STEC, EHEC, EIEC, EPEC, ETEC)	Enteric and diarrheal diseases, urinary tract infections, sepsis/meningitis	6619 confirmed cases of severe foodborne disease infections in EU/EEA in 2016	Ram et al. (2007), WHO (2018)
*Salmonella* species (mostly *Salmonella enterica*)	Salmonellosis, asymptomatic infection, gastroenteritis or typhoid fever	Global non-typhoid salmonella cases estimated for 200 million to 1.3 billion, with possible 3 million deaths a year in 2007	Coburn et al. (2007), Mumy (2014)
*Shigella* (*S. dysenteriae* is the most severe)	Shigellosis, gastroenteritis with dysentery	A total of 80 million cases occurred with 700,000 deaths a year, calculated in 2005	WHO (2005)
Enteroviruses (most significant Poliovirus)	Multiple, i.e. hand, foot and mouth disease, poliomyelitis, Bornholm disease, polio-like syndrome, pericarditis, myocarditis	A total of 57,628 cases occurred with 3,145 deaths and 21,269 paralysed patients, in 1952, in the United States	Zamula (1991)
*Vibrio cholerae*	Cholera	A total of 1,041,422 cases occurred with 9642 deaths in 1991 in America	Skowron et al. (2018)
*Clostridia* (mostly *C. perfringens*)	Type A food poisoning, necrotising enteritis, enterotoxaemias, bacteraemia, gas gangrene	Widely distributed in the soil and in faeces of humans and animals, dominant cause of food poisoning in the USA and Canada	Labbe and Juneja (2017), McClane (2014)
*Cryptococcus* (mostly *Cryptococcus neoformans*)	Meningitis, meningoencephalitis or disseminated disease	Major life-threatening fungal infection in patients with severe HIV infection, may complicate organ transplantation, reticuloendothelial malignancy, corticosteroid treatment or sarcoidosis	Kaplan et al. (2002)
Roundworms (mostly *Ascaris lumbricoides*)	Helminthiasis (incl. soil-transmitted): ascariasis, necatoriasis, cestodiasis, also malnutrition, anaemia, and others	Ascariasis classified as the most prevailing parasitic infection, about 1/5 of the world’s population affected	Amoah et al. (2018), Vieira Da Rocha et al. (2016)
*Giardia* (*G. lamblia*)	Giardiasis, severe diarrhoea	About 1/3 of the developing countries population affected, from 3 to 7% of people affected in the USA	Auerbach (2011)

Both the indirect and direct effects (see “Sewage sludge direct and indirect radiolysis” section) on pathogens caused by the EB are thought to result in the damage of DNA and RNA molecules. Nevertheless, the sensitivity of microorganisms to the accelerated electron beam varies significantly from one species to another. Decimal reduction dose (*D*_10_) is defined as the dose required for killing 90% of the microorganism population or the dose required for a one-log inactivation and is given by (van Gerwen et al. 2016)

4 $$ \log \frac{b}{b_0}=-\frac{1}{D_{10}}\ D $$

Where *b* is the number of surviving microorganisms, *b*_0_ is the initial number of microorganisms present and *D* is the dose absorbed by the microorganism. The measured *D*_10_ values for a number of microorganisms are shown in Table 7.

**Table 7:** *D*_10_ value of various microorganisms

Microorganism	*D*_10_ value (kGy)	Reference
*Absidia* sp.	≤ 6	Maruthi et al. (2013)
*Acinetobacter radioresistens*	1.3–2.2	van Gerwen et al. (2016)
*Ascaris lumbriccoides*	≤ 0.45	Maruthi et al. (2013)
*Aspergillus fumigatus*	0.6	Garcia et al. (1987)
*Aspergillus niger*	0.5	van Gerwen et al. (2016)
*Bacillus pumilus*	1.4 to 1.8	van Gerwen et al. (2016)
*Bacillus subtilis*	0.6	van Gerwen et al. (2016)
*Brucella abortus*	0.15	Somers (2004)
*Campylobacter* sp.	< 0.2	Somers (2004)
*Candida albicans*	0.9	Garcia et al. (1987)
*Clostridium botulinum*	1.4 to 4.2	van Gerwen et al. (2016)
*Clostridium difficile*	0.9	Garcia et al. (1987)
*Clostridium sporogenes*	1.6 to 2.2	van Gerwen et al. (2016)
*Clostridium tetani*	2.4	van Gerwen et al. (2016)
*Cryptococcus albidus*	2.7	Moreira et al. (2012)
*Cryptococcus laurentiii*	3.1 to 4.5	Maruthi et al. (2013), Moreira et al. (2012)
*Cryptococcus uniguttilans*	1.4	Moreira et al. (2012)
*Escherichia coli*	0.3–0.4	Borrely et al. (1998), Sommers and Boyd (2006)
*Klebsiella pneumonia*	0.12–0.28	Gautam et al. (2015)
*Lactobacillus brevis*	1.2	van Gerwen et al. (2016)
*Listeria monocytogenes*	0.62	Rajkowski (2016)
*Micrococcus radiodurans*	2.2	van Gerwen et al. (2016)
*Mycobacterium fortuitum*	0.6	Garcia et al. (1987)
*Mycobacterium tuberculosis*	0.3	Borrely et al. (1998)
*Pseudomonas* spp.	0.06	van Gerwen et al. (2016)
Poliovirus	1.85	Borrely et al. (1998)
*Saccharomyces cerevisiae*	0.5	van Gerwen et al. (2016)
*Salmonella muenster*	0.6	Garcia et al. (1987)
*Salmonella* sp.	0.6	Somers (2004)
*Salmonella typhimurium*	0.2 to 1.3	van Gerwen et al. (2016)
*Shigella dysenteriae*	0.6	Borrely et al. (1998)
*Staphylococcus aureus*	0.2–0.5	Somers (2004), van Gerwen et al. (2016)
*Streptococcus faecalis*	1.56	Garcia et al. (1987)
*Yersinia enterocolitica*	0.2	Somers (2004)
*Vibrio cholerae*	0.48	Borrely et al. (1998)

Determination of the applied dose for the sewage sludge treatment purpose is therefore related to microorganisms’ presence. Identification of the pathogen with the highest *D*_10_ allows the choice of the minimum radiation dose (lowest cost) that guarantees sufficient microbicidal effect.

## Technical aspects of sewage sludge electron beam irradiation

### Electron beam accelerator—principle of operation

Particle acceleration is an act of propelling the charged particles. For an electron beam accelerator, this particle is an electron which is negatively charged. There are three main components of the electron beam accelerator: electrons source, accelerating structure and delivery system (Hamm and Hamm 2012).

The electrons are released from a cathode via thermionic emission and the beam density is dependent on the temperature and cathode material properties (Zimek 1998). The cathode is usually the most crucial part of the electron source and its lifespan is defined by the cathode quality. After emission from the cathode, the electrons are accelerated towards the anode, which is positively charged, under the influence of a force (*F*_e_) created by the electric field (Zimek 1998):

5 $$ {F}_e=q\times {E}_{\mathrm{d}} $$

Where *q*—particle charge, for electron 1.602 × 10^−19^ [C]; and *E*_d_—density of electrical field [$\frac{V}{m}=\frac{N}{C}$].

The main difference amongst EB accelerators types is the method of electric field generation, and three main categories can be identified: high voltage direct current (DC), radio frequency (RF) and microwave linear accelerators (LINACs). Direct current acceleration involves putting the electrons through the voltage drop to give the particles the necessary velocity. There are many technical developments in this widespread EB category, but all of them require a high voltage supply (Chao and Chou 2017). The most important representatives of these category in relation to wastewater treatment are dynamitron, insulating core transformer (ICT) and coreless transformer (ELV) models. Radio frequency accelerators operate on the basis of the large, single volume called a resonance cavity, which is fed by radio waves (oscillating electromagnetic fields). There are two main installation within this category: the ILU- and Rhodotron-type devices. Excluding the ILU accelerator (also a linear device), LINACs are built from several small coupled copper cavities for resonating at microwave frequencies, and two main bands may be distinguished: L-band for 1–2 GHz and S-band for 2–4 GHz (Hamm and Hamm 2012). The cavities gain energy from the microwave generator, klystron or magnetron (Chao and Chou 2017). A detailed comparison can be found in Table 8.

**Table 8:** Comparison of accelerator types (Chao and Chou 2017; Cleland 2006; Hamm and Hamm 2012; Zimek 1998)

Accelerator type	Accelerator model	Principle of operation	Capability [MeV]
Direct current	Cockcroft–Walton	Capacitive, series-coupled	Up to 5
	Dynamitron	Capacitive, parallel-coupled	0.5 to 5
	Insulating core transformer (ICT)	Magnetic, series-coupled	0.3–3
	Coreless transformer (ELV)	Magnetic, parallel-coupled	0.2–2.5
	Van de Graaf	Positive charge-carrying belt	1–10
Radio frequency	ILU-type	One pass along the axis of the toroidal cavity	Up to 5
	Rhodotron-type	Multiple passes within the coaxial cavity	5–10
Microwave linear	LINACs	Several small coupled copper cavities	Up to 10

Current technical developments and trends in electron beam accelerators favour the more widely researched DC systems based on the well-proven ELV and Dynamitron technology. Moreover, smaller, more compact and self-shielded devices are desirable, if possible. The RF Rhodotrons and microwave LINACs are also being extensively developed, but they are significantly more expensive and space-consuming so their application for sewage sludge treatment only is not economically justified.

### Electron beam irradiation parameters

The numerous chemical parameters that affect the electron beam irradiation have been addressed in detail in the previous sections. However, there are also some technical features of the accelerator itself and the treatment process, which have implications for the implementation of the technology on the industrial scale. These are as follows (Borrely et al. 1998; Kurucz et al. 1991; Capodaglio 2017; Farooq et al. 1992; Hossain et al. 2018; International Atomic Energy Agency 2005; Kurucz et al. 1995; Nickelsen et al. 1994; Skowron et al. 2013; Trump et al. 1984; William et al. 2001):

- Accelerating voltage [V]
- Electron beam power [kW]
- Electron beam current [mA]
- Applied dose [kGy]
- Exposure time [s]

There are relationships between groups of these parameters. The applied voltage determines the energy of the accelerated electrons, which is usually expressed in MeV and 1 MeV = 1.602 × 10^-13^ J (Kurucz et al. 1991). The direct correlation between the power, beam current, and electron energy is usually described according to the following equation:

6 $$ P=U\times I $$

Where *P* is power [kW], *U* is the energy of the electrons [MeV] and *I* is the beam current [mA].

The electron beam dose depends on the beam power and the exposure time and is the key parameter upon which the pollutant decomposition efficiency depends. When relatively low accelerating voltage is implemented, the exposure time and/or the beam current has to be increased in order to maintain the efficiency of removal. The overall accelerator capital cost increases with both the accelerating voltage and the beam current (not to confuse with waste unit treatment cost). The running cost depends on power, as this determines the electricity requirement. The efficiency for converting electrical power into beam power depends on the accelerator technology but is in the range of 60–80% (International Atomic Energy Agency 2005).

The decrease in pollutant concentration during the treatment might be expressed exponentially as a first-order chemical kinetics reaction, using the formula (Takács et al. 2007; Wang and Chu 2016):

7 $$ X={X}_0\times {\mathrm{e}}^{- kD} $$

Where *X* (mol/l) is the solute concentration at the applied dose *D* (J/kg), *X*_0_ (mol/l) is the initial solute concentration and *k* (kg/J) is the dose constant representing the reaction rate, i.e. the amount of solute reduced per unit of the irradiation energy absorbed. It is possible to calculate the *k* constant from measurements, and it is related to the solution pH, the molecular structure of the pollutant, the water matrix, and the presence of some inorganic anions as well as radical scavengers (Le Caër 2011; Shin and Kang 2003; Takács et al. 2007; Wang and Chu 2016).

### Continuous slowing down approximation range of the electrons

**Fig. 2:**
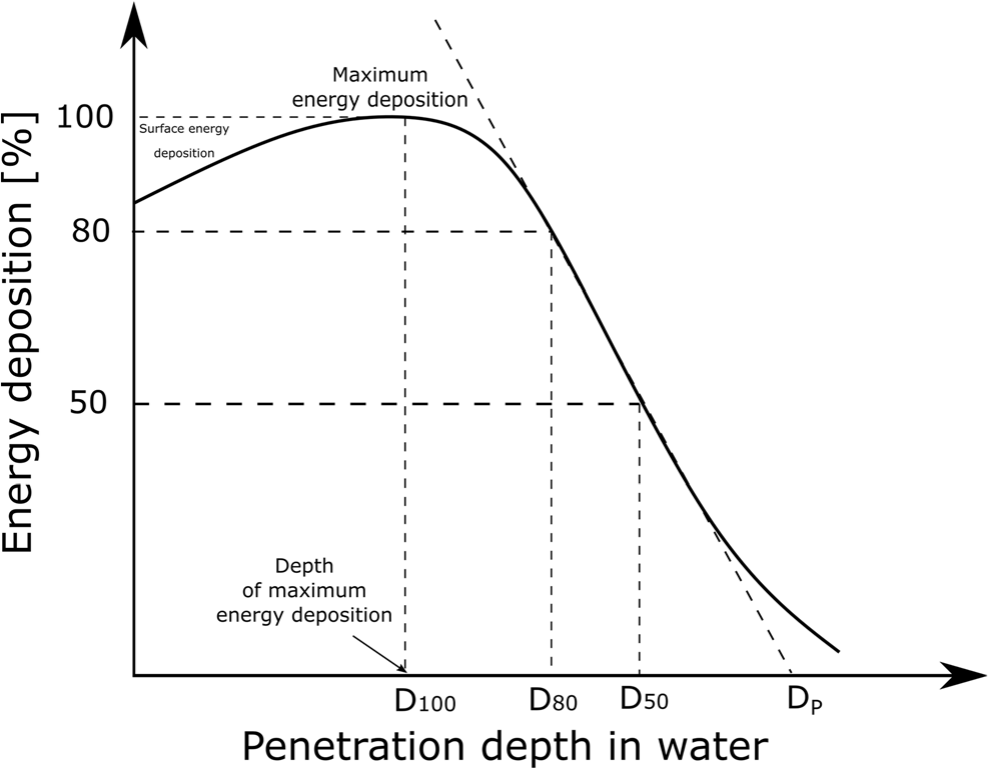
The energy deposition curve as a function of penetration depth, where *D*_100_ is the depth of maximum energy deposition, *D*_80_ is the depth of 80% energy deposition and D_P_ (or MPD) is the maximum penetration depth (based on Gann et al. 2004; Kurucz et al. 1995; Strydom et al. 2006)

The first consideration regarding an e-beam irradiation installation for both wastewater and sludge sterilisation is the energy of the applied electrons. To reduce the operation cost, it should be as low as possible (Sabharwal 2013). However, that energy determines the penetration depth of the electrons (or the range of the electrons). This parameter is critical when relatively high pollutant destruction effectiveness is required in one pass (William et al. 2001)**.** The shape of the energy deposition curve as a function of depth is presented below (Fig. 2). The electrons range defined as the continuous slowing down approximation range (CSDA) in pure water is shown in Table 9. Note that the maximum energy that can be used is 10 MeV, as above this, the electron beam can start to activate the material.

**Table 9:** CSDA range and total stopping power for electrons of various energies in pure water (20 °C) (data from the National Institute of Standards and Technology 2018)

The energy of the accelerator [MeV]	Total stopping power [MeV/cm]	CSDA range [g/cm^2^]
0.1	4.119	0.01
0.5	2.041	0.18
1	1.862	0.44
2	1.85	0.98
3	1.889	1.51
4	1.931	2.04
5	1.971	2.55
6	2.01	3.05
7	2.047	3.55
8	2.082	4.03
9	2.116	4.51
10	2.149	4.98

It is possible to determine the most beneficial depth of the sample for electron beam irradiation, where the value of absorbed energy reaches a peak (Fig. 2). The electron deposits its energy by interacting with atomic electrons (elastic and inelastic interactions; Krumeich 2018) and that loss depends on the energy of the electron and the time spent around the ion. Doses at the surface are relatively high, reaching 80–100% of the maximum energy absorbed (Strydom et al. 2006). Along with the energy loss, deceleration occurs, and time spent in the nearest area of nuclei increases, which results in a relatively larger energy deposition. However, as a significant amount of energy has been already lost, the absolute deposition is less. The optimal solution for these two contradictory processes can be seen as a peak in the energy deposition as a function of the sample depth. The maximum dose occurs at a specific distance called depth of maximum dose *D*_100_ (Fig. 2). Furthermore, since the approximate density of the water at the temperature of 20 °C is 1 g/cm^3^, the MPD of electrons [cm] may be determined directly from the CSDA [g/cm^2^] range plot. The relationship between the CSDA range, MPD and material density is given below:

8 $$ MPD=\frac{CSDA}{d} $$

Where *d* is the sludge density [g/cm^3^].

The higher the initial energy of the electron beam is, the deeper the dose can be delivered. When the CSDA range is expressed in the uniform unit of g/cm^2^, it only slightly depends on the type of material.

### The density of the irradiated material

In the energy range between 100 keV and 10 MeV, the maximum penetration depth is proportional to the beam energy but inversely proportional to the density of the material to be treated ( (8).

Since the sludge as well as the anaerobic digestate (Gerber and Schneider 2015) consists of solids and water, the content of total solids (TS) is the primary parameter upon which the average density of sludge depends. However, the density at the most stages of their treatment is similar to water (Fernandes et al. 2007; Sperling 2005) and typical values vary from 1.02 g/cm^3^ for liquid sludge to 1.1 g/cm^3^ for dewatered sludge (Fernandes et al. 2007; Sperling 2005). The MPD values for different sludge densities are shown in Table 10. Due to the small differences in density, there are only relatively small changes in the MPD, indicating that the same beam energy can be used for different types of sludge.

**Table 10:** Sludge density relation to total solids content in sludge (based on Fernandes et al. 2007)

Types of sludge	% TS	Density of sludge (g/cm^3^)		MPD [cm]			
				2 MeV		10 MeV	
		Min	Max	Max	Min	Max	Min
Primary sludge	2.0–6.0	1.003	1.01	0.98	0.97	4.96	4.93
Thickened mixed sludge	3.0–8.0	1.004	1.01	0.97	0.97	4.96	4.93
Primary thickened sludge	4.0–8.0	1.006	1.01	0.97	0.97	4.95	4.93
Digested mixed sludge	3.0–6.0	1.007	1.02	0.97	0.96	4.94	4.88
Secondary anaerobic sludge	3.0–6.0	1.01	1.02	0.97	0.96	4.93	4.88
Dewatered sludge	20.0–40.0	1.05	1.1	0.93	0.89	4.74	4.52

### General sewage sludge composition

As has been adduced before, there are many specific compounds in sewage sludge, which have a significant influence on the treatment process (free radical scavengers, PPCPs, POPs—“Free radical scavengers” section; hydroxide and oxonium ions—“pH influence” section). Nevertheless, there are also some general categories of sewage sludge components, reacting differently when undergoing the electron beam treatment.

**Fig. 3:**
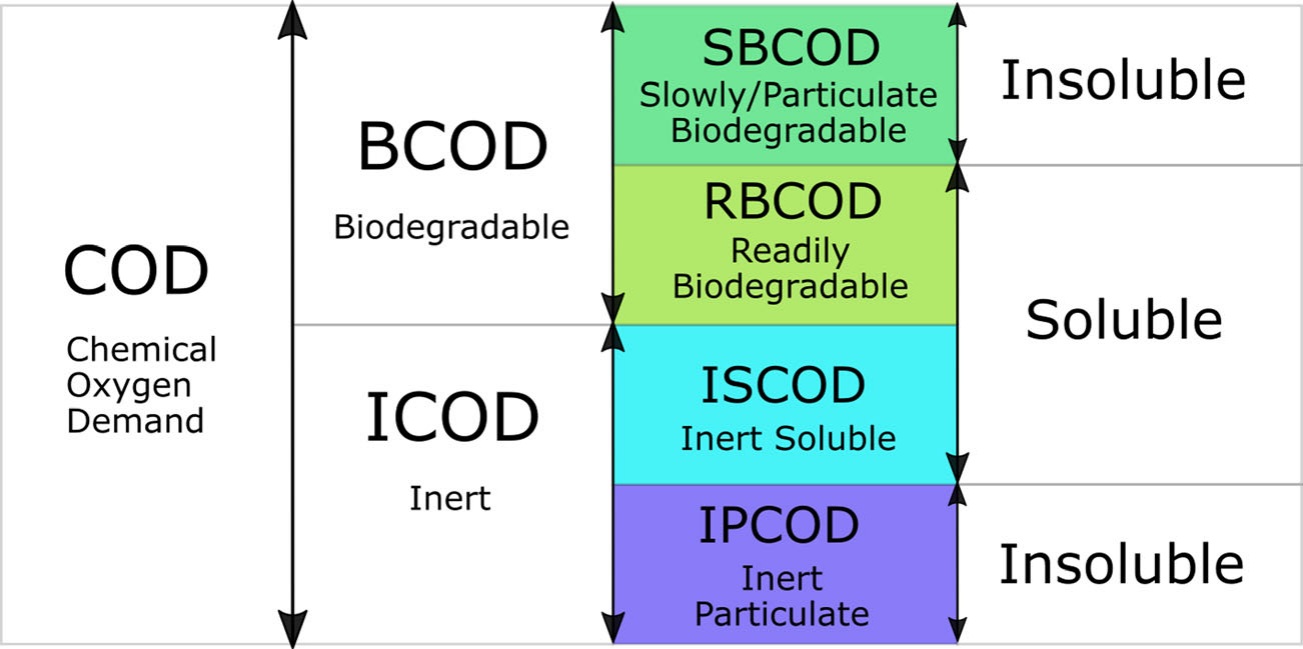
Distribution of chemical oxygen demand fractions in sewage sludge (based on Choi et al. 2017; Myszograj et al. 2017; Orhon and Cokgor 1997)

Using one of the most common wastewater quality indicators, chemical oxygen demand, the total organic matter can be divided into biodegradable (BCOD) and an inert (non-biodegradable) fraction (ICOD) (Orhon and Cokgor 1997). The biodegradable part can be further subdivided into readily biodegradable (RBCOD) and slowly/particulate biodegradable (SBCOD). The non-biodegradable fraction has two major components: soluble (ISCOD) and particulate (IPCOD). Therefore, the total COD can be represented accordingly (9, Fig. 3) (Choi et al. 2017; Myszograj et al. 2017; Orhon and Cokgor 1997):

9 $$ \mathrm{COD}=\mathrm{RBCOD}+\mathrm{SBCOD}+\mathrm{IPCOD}+\mathrm{ISCOD}\left[\mathrm{g}{O}_2/{m}^3\right] $$

The RBCOD refers to matter that can be quickly and easily degraded by microorganisms, like volatile fatty acids or leachate from municipal landfills, whereas SBCOD decomposition is slow, caused by various microbial metabolism processes (Choi et al. 2017). Both RBCOD and SBCOD have to be hydrolysed prior to destruction (Myszograj et al. 2017). The IPCOD fraction is insoluble and impossible to decompose by microorganisms, but it is effortlessly removable by sedimentation, while the ISCOD poses a serious challenge to traditional WWTPs as it usually means the presence of such persistent pollutants as organic, aromatic compounds (benzene, toluene, other substances with a benzene ring).

Multiple and sometimes inconsistent information have been reported regarding the EB influence on both BOD and COD of the sewage sludge. Many researchers reported the COD to be only slightly changed after the irradiation (Farooq et al. 1992; Kim et al. 2007; Zheng et al. 2001), while others noted significant growth in soluble COD parameter (Changqing and Min 2012; Park et al. 2009), reaching 2400% increase, when the EB at 6 kGy (1 MeV ELV-4 Model, Pyrex tray 35 × 22.5 × 5.6 cm) was applied as the sludge anaerobic digestion pretreatment (Shin and Kang 2003). Nonetheless, the considerable changes were only observed in the SCOD part. Therefore, it can be indicated that using the EB, the insoluble part of the COD can be converted, increasing the SCOD contribution but not changing the overall COD value.

It is not possible now to explicitly determine the EB influence on BOD as there are both reports on its increase (Kim et al. 2007; Paul et al. 2011) and decrease (Han et al. 2012; He et al. 2016), and this issue should be investigated further.

### Sewage sludge thickness

**Fig. 4:**
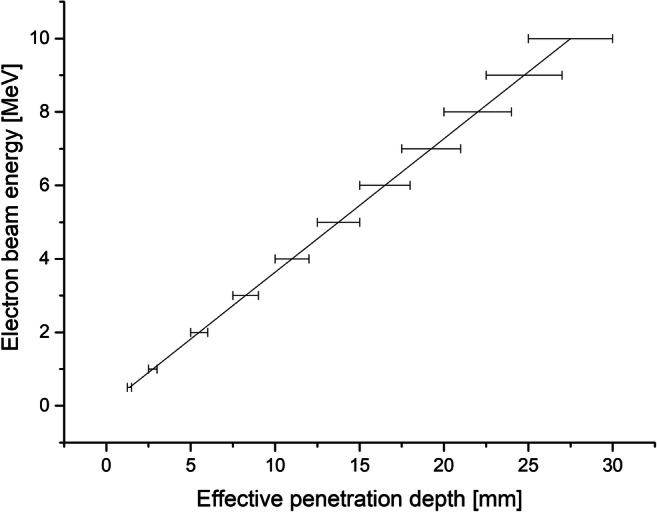
The effective penetration of the electrons into the sewage sludge within the 1-10-MeV range (Alcántara and Cruz 1997)

Given the fact that the ability of electrons for sludge matrix penetration is one of the most important parameters on which e-beam system efficiency depends, the optimal technical solution of sludge delivery system should be implemented. Therefore, it requires an accordingly thin layer of waste to be irradiated (Fig. 4). Various options of such technology have been examined so far, both continuous and batch. The summary of the layouts reported so far can be found in Table 11.

**Table 11:** Sewage sludge irradiation layouts reported so far. Best results, if determined, *are* presented in *italics*

Process layout	EB energy [MeV]	Treatment dose [kGy]	Sludge thickness [cm] and/or throughput [per min]	Reference
Batch	1.8	2–20	0.3–1 (*0.5*)	Changqing and Min (2012)
	1	0.5–*6*	5.6	Shin and Kang (2003)
	1	1–*7*	*0.25*–1	Park et al. (2009)
	10	0.2–*10*	Not determined, probably < 0.5 cm^a^	Praveen et al. (2013)
	10	1–*10*	Not determined, probably < 1 cm^b^	(Skowron et al. (2013)
Continuous sludge cascade	3	2.7–30.7	Not determined, sludge flow 113 l/min	Engohang-Ndong et al. (2015)
	1.5	4	~ 0.38, sludge flow 460 l/min	Kurucz et al. (1995), Waite et al. (1985)
	1.5	4	Not determined, sludge flow 450 l/min	Trump et al. (1984)
Continuous, conveyor belt, with the nozzle-type injector	2.5	10	0.7, sludge flow 8.3 kg/min	Kim et al. (2009)
	1	1–3	0.4, sludge flow 40 l/min	Shin et al. (2002)
	10	5–*7*	2.5-3, sludge flow 48.6 kg/min	Chmielewski et al. (1995)
	10	5–15	Not determined, sludge flow 67.4 kg/min	Mckeown (1996)
	2	2–5	0.1–1, 5 kg/min	Takeshita and Naramoto (1992)

^a^Samples triple bagged in Whirl-Pak® bags (12.5 cm L × 7.5 cm W, 0.057 mm), 20 ml of sludge each

^b^Samples bagged in plastic, synthetic bags, 100 ml of sludge each

**Fig. 5:**
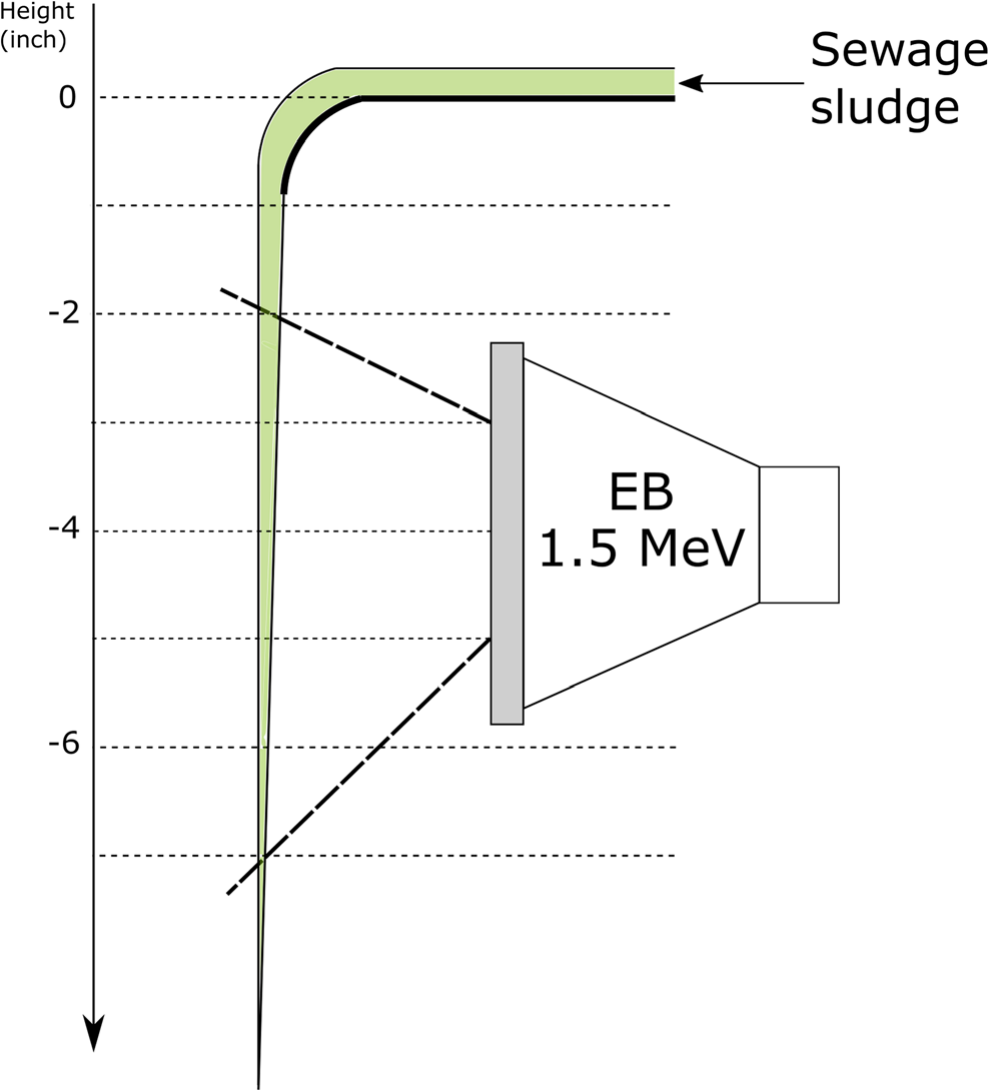
The scheme of the cascade-type sludge sterilisation system (based on Waite et al. 1985). Cross-section of the sludge stream falling over the weir

**Fig. 6:**
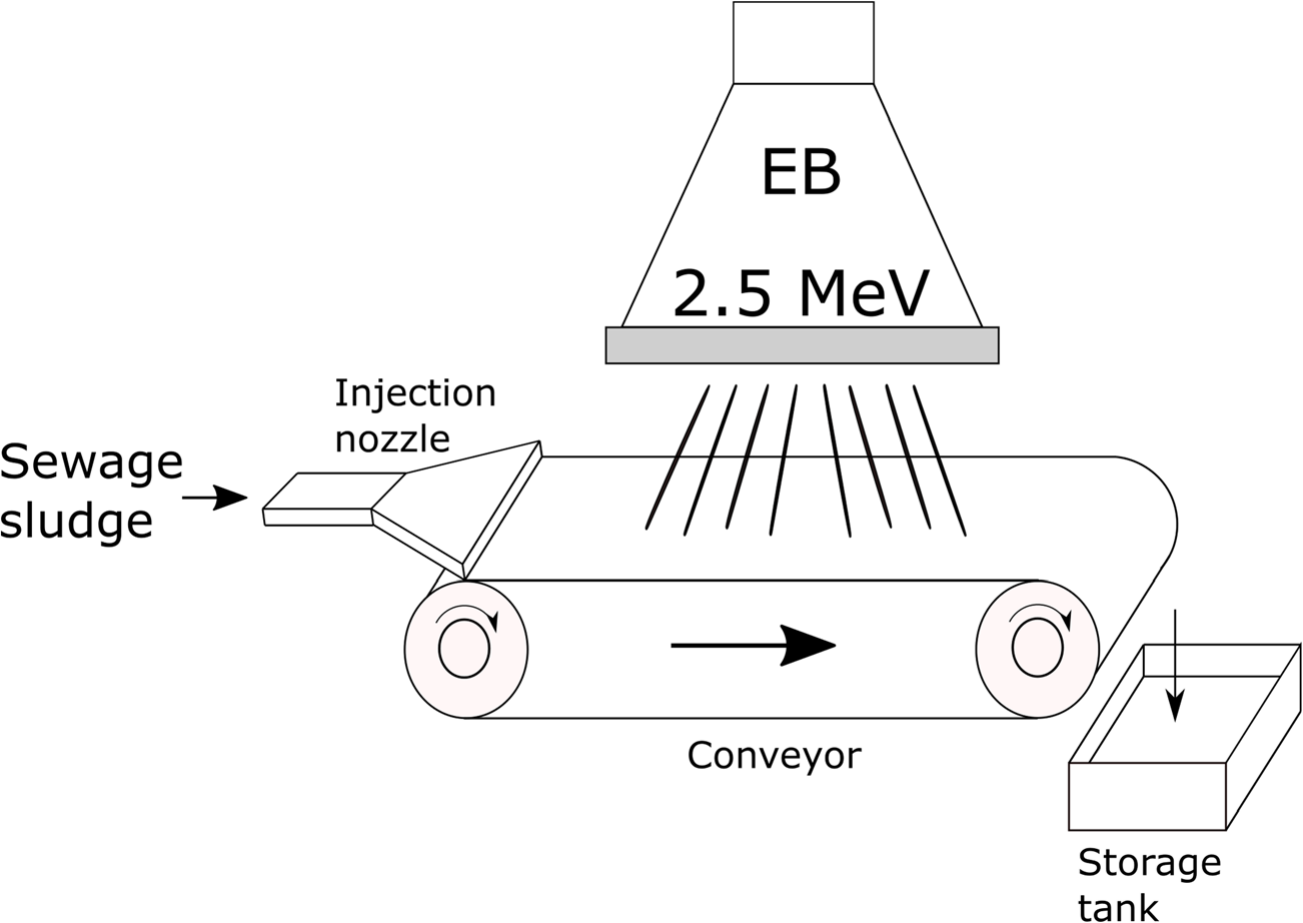
The scheme of the nozzle-type sludge sterilisation system (based on Kim et al. 2009)

**Fig. 7:**
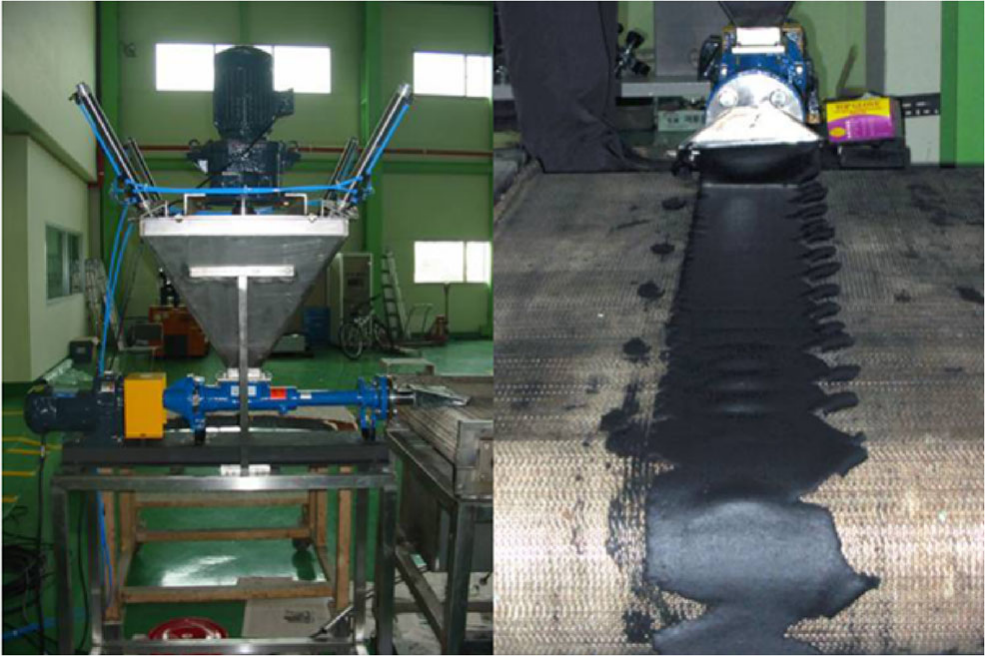
The picture of nozzle-type sludge sterilisation system. Image courtesy of Kim et al. (2009)

Batch layout was usually applied during laboratory tests, and various sludge thickness options were provided in accordance to the available accelerator. In general, there have been two different ways of continuous sludge delivery system so far: sludge cascade using gravity (Engohang-Ndong et al. 2015; Kurucz et al. 1995; Trump et al. 1984; Waite et al. 1985) (Fig. 5) and a mechanical conveyor belt, usually with the nozzle-type injector (Kim et al. 2007, 2009; Mckeown 1996; Shin et al. 2002) (Fig. 6 and Fig. 7). It can be clearly seen that the crucial factor during the low-energy EB application is material thickness as the MPD in pure water for 1 MeV is only 0.44 cm. Nevertheless, the effective penetration of the electrons into the sewage sludge is claimed to be only about 2.5–3.0 mm per 1 MeV (Alcántara and Cruz 1997) due to the presence of several scavengers, and the relationship between the sludge thickness and energy can be introduced linearly as (10, Fig. 4):

10 $$ y=0.3636x $$

Where *y*—energy of the accelerator within the range 1–10 MeV and *x*—effective penetration depth/required sludge thickness [mm].

When a high energy accelerator might be used (MPD around 5 cm for 10 MeV), the treatment dose becomes critical since electrons can penetrate a large volume of sludge and high-dose application ensures desired sterilisation effect. The average dose absorbed by the material with the low atomic number (*Z*) can be described as (Zimek 1998):

11 $$ D=\frac{E\times j\times t\times {10}^6}{\mathrm{CSDA}} $$

Where *D*—dose [Gy], *E*—electron energy [MeV], *j*—beam current density [mA/cm^2^], *t*—time [s] and CSDA—penetration range [g/cm^2^].

There were various doses tested for sludge sterilisation so far, ranging from 1 up to above 30 kGy (Table 11), but the 10-kGy absorption is enough for sludge to meet the U.S. EPA Guideline Class A Biosolids requirements (Kim et al. 2009; US EPA 2003).

### Cost–benefit analysis and dose optimisation

One of the most important factors to consider while designing any industrial process is cost investigation for both capital and operational expenditure. Detailed analysis for various acceleration parameters is presented below (Table 12).

**Table 12:** Operational and capital costs for various EB treatment parameters (adapted from Han et al. 2012; Kim et al. 2009; Pillai and Reimers 2013)

Accelerator type	Dry solids content [%]	Capacity [TDS/year]	Capacity [m^3^/day]	Operating cost [$/TDS]	Capital cost [$]
Dual, ~ 2 mA, 18 kW, 10 MeV, 15 kGy	2.6	7875	28.63	378	15 million
	4.3	11,250	38.78	232	
Duala, 100 mA, 50 kW, 15 kGy	2.6	393,750	1332	6.79	
	4.3	562,600	1927	4.75	
40 mA, 100 kW, 2.5 MeV, 10 kGy	18–20	15,660	226	4.4	1.98 million
		33,556	484	2.2	
400 mA, 400 kW, 1 MeV, 1 kGy	N/A		10,000	0.3 per m^3 b^	4 million

^a^Theoretical calculation, scaled-up from 2 mA scenario (Pillai and Reimers 2013)

^b^Textile wastewater treatment plant operating in Daegu Dyeing Industrial Complex in Korea. Operating cost per m^3^ instead of per TDS as there are mostly liquid impurities present in textile waste (Han et al. 2012)

Despite the fact that literature lacks many examples of industrial-scale EB sludge treatment facilities, the throughput and related cost might be relatively easily scaled up for a certain accelerator type, when cost is only dependent on electricity consumption. As the EB system irradiation dose rate is directly related to beam current (11), higher currents ensure certain dose delivery in a shorter treatment time, allowing for increased waste load throughout the year and decreased cost. It can be clearly seen that for EB treatment both operating and capital costs per waste unit decrease with capacity increase, and therefore, treatment of a small amount of sludge is not competitive with currently existing methods, but the 100-mA (with 50 times higher dose rate compared to 2 mA) option is much less expensive (Table 13) than any other traditional system. There is no additional treatment (such as AD) required for 15 kGy dose (Pillai and Reimers 2013).

**Table 13:** Comparison of the EB water radiolysis with currently available methods for sewage sludge treatment

Parameter	Unit	Anaerobic Digestion	Electron beam + AD	Thermal hydrolysis + AD	Incineration with energy recovery
Energy yield	kWh/TDS	820	1550–2353^a^	1100	880
Solids destruction	%	34	30–60^a^	45	77
Carbon emission	kg CO_2_/TDS	593	Not modelled	143	2000–4000^b^, with additional N_2_O and NO_*x*_ emissions
Eco-friendliness	Low to high	Moderate	High	Moderate	Low
Sludge usage as fertiliser	Yes/no	Yes	Yes	Yes	No
Treatment cost (2005)	£/TDS	200-345	55–145^c^	345–630	290–690
HRT	Days	20–30	10–15^1^	10–15	N/A
Footprint	Small to large	Medium Aerobic digestion tank	Medium Aerobic digestion tank + self-shielded accelerator/ irradiation chamber + CHP unit	Large Aerobic digestion tank + pulper, reactor and flash tank + CHP unit	Large Incineration chamber
Sludge delivery system	Uncomplicated/complicated	Uncomplicated	Complicated	Uncomplicated	Uncomplicated
Removal level of bacteria, viruses and pathogens	Log reduction	2	> 5	4	Not modelled, potentially high
Antibiotic resistance genes removal	%	Up to 50.7	Not modelled, potentially high, further study required	Up to 52.5	Not modelled, potentially high
Pharmaceuticals removal	%	7–99; limited data available	50–99 (without the AD unit), limited data available, lack of information on transformation products	Up to 99, limited data available, metoprolol and progesterone can be enhanced during process	Not modelled
Colour removal	%	60–87 (20–30 days)	90–99 (without the AD unit)	Colour is formed during thermal hydrolysis process	Not modelled
Persistent organic pollutants	%	25–80	70–99 (without the AD unit), limited data available	Not modelled, limited data available, 55.5 reported for paranaphthalene	Not modelled
Heavy metal removal	%	25 to 96; e.g. 90–96% for less soluble metals like aluminium, < 50% for dissolved mercury	10–100 (without the AD unit), 10 to 30% while using the EB only or in up to 99% if some hydroxyl radical scavenger is applied	Not modelled, limited data available	2-55
Microplastics removal	%	Common plastics are not biodegradable, effluent from sewage treatment is thought to be a major source of microplastics to freshwater bodies	Not modelled, some common plastics are reported to interact with the electron beam	Not modelled, some plastics are reported to undergo changes at THP conditions	Not modelled, common plastics are usually decomposed in up to 600 °C
Pretreatment	None/requirement	Requires sludge to be heated (approximately 37 °C) and thickened	Requires sludge thickness to be very thin, preferably up to 1 cm	Requires centrifuge thickening to 16–18% DS	Advanced mechanical dewatering process needed
Hazardous chemicals and by-products production	No to possible	Not likely	Possible, further study required	Possible, potential for production of refractory material and higher ammonia concentration than standard AD	Possible, PAHs and PCBs emitted to the atmosphere
Technology readiness	Existing/developed	Existing, numerous sites successfully operating at full-scale	Developed, some pilot and commercial sludge treatment plants operating	Existing, numerous sites successfully operating at full-scale	Existing, numerous sites successfully operating at full-scale
Technical problems	Description	Installation of considerable size with low efficiency, limited biodegradability of sludge without additional process variations	Penetration depth limit—sludge delivery system	Large footprint, involves using high temperature (165 °C) and pressure (6 bar), sludge needs cooling prior to anaerobic digestion	CO_2_, N_2_O and NO_*x*_ emission handling, large footprint, involves using high temperatures 1200–800 °C
References		Andrady (2011), Cavinato et al. (2010), Fu et al. (2018), Gardner et al. (2013), Horton et al. (2017), Mahon et al. (2016), Mills (2015), Phan et al. (2018), Varel et al. (2012), Wang et al. (2019), Wei et al. (2019a, 2019b), Zhang and Chen (2020)	Bae et al. (1999), Capodaglio (2017), Changqing and Min (2012), Chmielewski et al. (1995), Engohang-Ndong et al. (2015), International Atomic Energy Agency (2008, 2005), Kim et al. (2007, 2009), Kurucz et al. (1995), Lock et al. (2010), Malmborg and Magnér (2015), Mckeown (1996), Nickelsen et al. (1994), Park et al. (2009), Paul et al. (2011), Pikaev et al. (1997), Pillai and Reimers (2013), Shin and Kang (2003), Skowron et al. (2018), Tobien et al. (2000), Trump et al. (1984), Waite et al. (1985), Wang and Chu (2016), Yao et al. (2005)	Barber (2016), Dwyer et al. (2008), Laurent et al. (2011), Levantesi et al. (2015), Malmborg and Magnér (2015), Mills (2015), Mocé-Llivina et al. (2003), Wang et al. (2019), Yang et al. (2017), Zhorin et al. (2018), Zhou et al. (2015)	Chen and Yan (2012), Dyke et al. (2003), Johnke (1996), Lin et al. (2018), Liu et al. (2010), Mills (2015), Mladenović et al. (2016)

^a^When waste activated sludge irradiated at 1–6 kGy, 1 MeV accelerator (Shin and Kang 2003)

^b^In the MSW incinerator, municipal sludge, 30% dry solids content (Johnke 1996)

^c^For the electron beam system only, 50 kW, 100 mA, 15 kGy delivery

### Accelerator operation

The existing accelerator technology is not able to operate continuously, as time is required for maintenance and repairs. Most current technology can be used reliably for 16 h a day, 20 days per month. This means that the sewage sludge must be stored before treatment. In most plants, this will not cause any problems as storage already takes place. The technology used in the accelerators is constantly being improved and the time for which the accelerator can be operated and the reliability is being increased.

## Electron beam comparison with other techniques

### Electron beam comparison with AD, THP and incineration

Currently, around 84% of sewage sludge in the UK is treated by anaerobic digestion (Ofwat 2016). This technology is based on the breakdown of organic material by microorganisms in the absence of oxygen, and it has an additional advantage of producing biogas, which can be transformed into electricity and fuel in the combined heat–power plant (Mills 2015). However, anaerobic digestion is limited by relatively slow dry solids hydrolysis, and it requires large environmental footprints (Xue et al. 2015). Therefore, several improvements have been developed to make the anaerobic digestion more efficient and beneficial. The most notable of these is thermal hydrolysis process (THP). It is also common to use incineration with energy recovery, where temperatures range between 800 and 1200 °C (DEFRA 2012). There were some tests on implementing the EB before the AD unit (Park et al. 2009; Rawat and Sarma 2013; Shin and Kang 2003), but most of the studies have been done on EB radiolysis of the already anaerobically treated sludge. The list of the most important parameters of the AD itself, EB + AD and THP + AD and of the incineration processes is shown in Table 13.

### Removal of bacteria, viruses and pathogens

The main purpose of the sewage sludge treatment is to remove the pathogenic microorganisms so that it can be safely treated as substrate (usually fertiliser), landfilled or stored (Świerczek et al. 2018). The overall efficiency of this hygienisation for the AD, THP and EB is 99% (2 log reduction), 99.99% (4 log reduction) and > 5 log reduction for 10 kGy EB, respectively (Chmielewski et al. 1995; Chmielewski and Sudlitz 2019; Levantesi et al. 2015; Mills 2015; Taboada-Santos et al. 2019).

Although all of the methods have very good performance, the mechanism of pathogen removal is different for each of them. AD treatment is based on the combination of competitive microbial interactions, time spent in the reactor and temperature influence as well as build-up or presence of toxic metals (Appels et al. 2008). The pathogens’ die-off kinetic models for these parameters are well-known and widely described in the literature (Avery et al. 2014). THP reduction in hazardous microorganisms is based on high temperature and high pressure which are applied simultaneously and are detrimental to the biological cells of pathogens and their proliferation functions, significantly increasing treatment efficiency when compared to AD only (Levantesi et al. 2015). EB hygienisation, although affected by several similar parameters like toxic metal presence, induces microorganism destruction by high energy electron penetration into the sludge and the production of radicals (see “Sewage sludge direct and indirect radiolysis” and “Influence of the presence of pathogens” sections). The incineration process involves converting sludge into gases, water and ash by extremely high temperature, which is lethal for all pathogen types but has been recognised as no longer a viable option for waste management (Greenpeace 2019).

There are multiple organisms reported to be resistant to AD and THP, and some pathogens can be enriched, but THP treatment is able to meet the class A biosolids requirements (Mocé-Llivina et al. 2003; Sassi et al. 2018; Wang et al. 2018; Zhao and Liu 2019). It is worth noting that the EB pathogen removal capability is only dependent on the applied dose (Table 7), and there is no organism resistant to irradiation present in the literature. Summary of the AD, THP and EB efficiency in the removal of several microorganisms is presented in Table 14.

**Table 14:** Compilation of log drop for different types of treatment

Pathogen	AD	Reference	THP	Reference	EB (10 kGy)^a^	Reference
Total coliforms	0.3–3	Avery et al. (2014)	Unknown	-	4	Chmielewski et al. (1995)
Faecal coliforms	1.3–3.0		4^b^	Wang et al. (2018)	> 8	Chmielewski et al. (1995)
Enterococcus	No change–1.6		> 2.70		> 8	van Gerwen et al. (2016)
*C. perfringens*	No change		0.3–2.5c	Carrington (2001)	> 8	Kim et al. (2018)
*E. coli*	1–2		3.2–5.3	Levantesi et al. (2015)	> 8	Borrely et al. (1998)
*Salmonella*	0.2–2.23		0.9–2.3	Levantesi et al. (2015)	> 8	Garcia et al. (1987)
Protozoa *Cryptosporidium*	0.3		> 4	Blewitt (1989)	> 2	Demirci and Ngadi (2012)
*L. monocytogenes*	2.23		> 4^d^	Environment Agency (2003)	> 8	Rajkowski (2016)
*C. jejuni*	− 1.0		> 5d	Environment Agency (2003)	> 8	van Gerwen et al. (2016)
Enteroviruses	No change–2.0		> 4	Astals et al. (2012)	2–2.9	Demirci and Ngadi (2012)
Somatic coliphage	0.09		3.9–5.2	Levantesi et al. (2015)	2.5	Praveen et al. (2013)
Poliovirus	6.2		Unknown	–	4.4–5.4	Borrely et al. (1998)
Protozoa *Giardia*	No change–2.0		> 4	Blewitt (1989)	> 2	Lenaghan (2008)
*Ascaris suum* ova	No change		> 3c	Carrington, (2001)	> 8	Capizzi-Banas and Schwartzbrod (2001)

^a^Data extrapolated for the dose required for meeting the class A biosolids efficiency (Kim et al. 2009; US EPA 2003)

^b^Assuming that the faecal coliform concentration in untreated sewage sludge is ~ 10^6^ microorganism per 100 ml (Kim et al. 2009)

^c^Assuming that THP complies with the class A biosolids requirements (Wang et al. 2018)

^d^Assuming that THP has better performance than pasteurisation in 70 °C for 1 h (Pickworth et al. 2012)

### Electron beam comparison with other irradiation techniques

As mentioned, irradiation by electron beam for sludge hygienisation has been introduced several decades ago. Nonetheless, the ionising radiation for such treatment is not only limited to acceleration techniques (Priyadarshini et al. 2014). There are several other techniques with a similar principle of treatment and method efficiency, which uses different irradiation sources, such as alpha, beta, gamma and X-ray. Amongst them, only the gamma installations have been implemented on the industrial scale (Rathod et al. 2009). There are many gamma irradiation plants successfully operating all over the world, accounting for the majority of facilities using the ionising radiation for liquid wastewater treatment purposes (Asgari Lajayer et al. 2019).

Gamma radiation, also known as γ rays, refers to short wavelength, high-frequency electromagnetic radiation of very high energy (photons with no charge and rest mass). It is emitted by unstable nuclei during its transition to a lower-energy state. Given the nature of γ rays as photons, they are also the most penetrating amongst other types of electromagnetic radiation and thus the most biologically hazardous (Freita-Silva et al. 2015). A gamma irradiation system involves using radioactive (self-disintegrating) isotopes, and amongst hundreds of gamma emitters, only cobalt ^60^Co or caesium ^137^Cs is permitted for radiation processing (da Silva Aquino 2012).

Currently, ionising radiation techniques for sludge treatment are only considered through gamma and electron beam irradiation. Both systems, although inducing water radiolysis and consequently highly reactive radical formation, have great differences that should be considered prior to irradiation treatment plant design. The up-to-date list of known γ rays and EB features can be seen in Table 15.

**Table 15:** Ionising radiation techniques for sludge hygienisation comparison: electron beam versus gamma rays (Freita-Silva et al. 2015; GIPA and iia 2017)

Parameter	Gamma irradiation	Electron beam
Irradiation doses	Low to medium, cannot be quickly adjusted	Medium to high, can be quickly adjusted
Processing timea	Slow, typically < 24 h	Fast, typically < 8 h
Operation	Inflexible, cannot be turned off; gamma rays emitted in all directions	Flexible, can be turned off; electrons aimed directly at the product
Penetration	Excellent, high efficiency for all product types	Low, efficiency depends on product density
Eco-friendliness	Low, uses radioactive material that needs proper disposal, necessary radionuclides are produced by nuclear reactor	High, uses electricity to generate high energy electrons
Operation cost	Low, requires additionally gamma rays source replacement and disposal annually	Low, greater demand for electricity than gamma installation
Capital cost	Medium, cost of irradiation source, conveyor + safety/control systems, specific and common infrastructure	High, cost of irradiation source higher than for gamma rays, conveyor + safety/control systems, specific and common infrastructure

^a^When the same volume of product, typical 45-ft tractor trailer (~ 85 m^3^) is treated (GIPA and iia 2017)

The main differences about the EB and gamma systems are penetration possibilities and irradiation generation. Due to high energy, no charge and no mass, photons can easily penetrate most of the waste both solid and liquid, while electrons can be relatively easily attenuated and dispersed by high-dense matter (see “Continuous slowing down approximation range of the electrons” section). Therefore, the material to be irradiated has to be accordingly thin for EB treatment, while gamma source can penetrate as far as 120 cm of 0.4 g/cm^3^ dense product (GIPA and iia 2017). Nonetheless, despite this undeniable advantage of gamma ray over EB accelerator, usage of a radioactive source, its handling and safe disposal should be considered very carefully. Electron beam technology has been improved significantly throughout the last 20 years, and it is no longer a highly expensive equipment, only available for limited use (Hossain et al. 2018). Therefore, given the current worldwide trends towards more eco-friendly, reliable and long-lived installations, it is advised to avoid technologies dependent on highly biological hazardous components, when possible (Cordella and Kaps 2018). Nevertheless, usage of gamma rays for other industries that benefit from radiation processing including medicine, food, polymers and automotive industries might be more justified due to the high-density material to be irradiated.

## Conclusions

The following can be concluded from the literature:

- A microbicidal action of ionising radiation is achieved by its direct (physical) and indirect (chemical) action. Direct interaction with the organic compound may only be significant when the concentration of the contaminant is ≥ 0.1 M. The indirect effect of irradiation is the result of free radicals forming in water that can interact with each other and with the molecules of the pollutant.
- The sensitivity of microorganisms varies significantly from one species to another. Hence, identification of the pathogens existing in the sludge to be irradiated and their lethal doses are necessary when designing the EB treatment facility. The pathogen with the highest *D*_10_ should be determined.
- In real conditions (sewage sludge, wastewater/natural water with a complex composition), some components naturally found in water scavenge reactive chemical species produced during treatment (e.g. O_2_, HCO_3_^-^, CO_3_^2−^ , Cl^−^, NO_2_^-^, NO_3_^-^) both decreasing (more likely) or increasing (less likely) overall process efficiency.
- In some cases, the addition of the non-water origin scavenger may lower the dose (cost) requirement by increasing the overall efficiency of the EB irradiation process.
- The solution pH has a noteworthy influence on the main water radiolysis products and their initial yields. Degradation of pollutants is favoured at weak acidic to neutral conditions. Thus, controlling of solution pH is a parameter, which may relatively easily increase the electron beam treatment efficiency and further investigation of this issue is recommended.
- The most important reactive species amongst water radiolysis products responsible for contamination removal should always be identified.
- For the EB influence on the BOD and COD parameter determination, it is necessary to investigate the content of the microorganisms and the biodegradable and inert part of the sludge as it can be critical to fully understand the chemical action of the reactive species created by accelerated electrons.
- Since such chemicals like hormones or pharmaceuticals can not only react with reactive species but also be a source of toxic by-products (transformation products), further examination of their reactions with electron beam origin radicals is highly recommended.
- Within the energy range between 100 keV and 10 MeV, the maximum penetration depth is inversely proportional to the density of the material to be irradiated. It is claimed that the penetration of the electrons into sludge matrix is only 2.5–3 mm per 1 MeV due to the presence of distorting compounds and differences in total solids content. However, due to the small differences in density, there are only minor changes in the MPD, indicating that the same beam energy can be used for different types of sludge.
- The electron beam facility needs to be designed with sufficient electron beam power and high electron beam utilisation capacity, as well as high accelerator electrical efficiency, to reduce unit operation cost and increase productivity along with possibly low electron energy for capital and operation cost reduction.
- The most important benefits of the electron beam sewage sludge treatment system are the possibility of removing various pollutants at the same time by both oxidation and reduction, low exploitation costs, the eco-friendly character of technology and the unit cost decrease along with the increase of the throughput and sludge biodegradability improvement. The most important disadvantages of the electron beam sewage sludge treatment are electron penetration limit and appropriate material distribution.
- Sludge sterilised with the e-beam can be used as soil fertiliser immediately after treatment, and no large land areas are needed for disposal for long periods.
- It is necessary to develop a normalised testing procedure and standardised requirements to enable cross-referencing between research facilities.

## Nomenclature

AD: anaerobic digestion
ARP: advanced reduction process
BCOD: biodegradable COD
BOD: biochemical oxygen demand
COD: chemical oxygen demand
CFU: colony forming unit
CSDA: continuous slowing down approximation range
DDIC: Daegu Dyeing Industrial Complex
DNA: deoxyribonucleic acid
DOC: dissolved organic carbon
DS: dry solids
EB: electron beam
EBRF: Electron Beam Research Facility
HM: heavy metal
HOC: halogenated organic compound
HRT: hydraulic retention time
ICOD: inert COD
IPCOD: inert particulate COD
IR: ionising radiation
ISCOD: inert soluble COD
MPD: maximum penetration depth
PAH: polycyclic aromatic hydrocarbon
PCB: polychlorinated biphenyl
POP: persistent organic pollutant
PPCP: pharmaceutical and personal care product
RBCOD: readily biodegradable COD
RNA: ribonucleic acid
SBCOD: slowly/particulate biodegradable COD
SCOD: soluble COD
THP: thermal hydrolysis process
TS: total solids
WWTP: wastewater treatment plant
